# Anatomical and Functional Outcomes of Human-Amniotic Membrane Graft in Refractory Macular Hole Cases

**DOI:** 10.3390/vision9020045

**Published:** 2025-05-22

**Authors:** Soefiandi Soedarman, Sandi Muslim, Waldensius Girsang, Elvioza, Referano Agustiawan, Alberthus Donni Budi Prasetya, Ichsan Fauzi Triyoga

**Affiliations:** 1 JEC Eye Hospitals & Clinics, Jakarta 10310, Indonesia; waldensius@jec.co.id (W.G.); elvioza@jec.co.id (E.); referano@jec.co.id (R.A.); 2 JEC Eye Hospitals & Clinics Candi @Semarang, Semarang 50149, Indonesia; sandi.muslim@jec.co.id; 3 JEC Eye Hospital & Clinics Primasana @Tanjung Priok, Jakarta 14320, Indonesia; alberthusdonni17@gmail.com; 4 Faculty of Medicine, University of Indonesia, Jakarta 10430, Indonesia; ichsnfzi@gmail.com

**Keywords:** refractory macular hole, human amniotic membrane (hAM) graft, amniotic membrane transplantation (AMT), macular surgery

## Abstract

Macular hole (MH) surgery generally has a high success rate, but finding anatomical plug for refractory cases remains challenging. The human amniotic membrane (hAM), with its anti-inflammatory and regenerative properties, has emerged as a potential option. This study aims to report the anatomical and functional outcomes of human amniotic membrane (hAM) graft as an intervention to repair refractory macular hole cases where wide internal limiting membrane (ILM) peeling was unsuccessful. A retrospective chart review was conducted at a single center, with the main outcomes being closure rate and postoperative BCVA at 6 months. Eleven eyes of 11 patients with refractory macular holes were identified and included in the study. Participants were predominantly males (72.73%) with a mean age of 49.27 years. Nine eyes achieved successful MH closure with a single intervention and showed no recurrence during the 6-month follow-up. Mean BCVA at 3 and 6 months improved significantly (*p* = 0.0207) from 1.747 ± 0.74 logMAR to 1.210 ± 0.51 logMAR and 0.939 ± 0.47 logMAR (range 2.079–0.301 logMAR). The use of human amniotic membrane (hAM) graft seems to be a viable and effective alternative for the treatment of refractory macular holes. However, further larger prospective controlled studies are necessary to confirm our results.

## 1. Introduction

The failure rate of primary surgery in idiopathic macular hole (MH) is less than 10% [1]. Following primary vitrectomy with ILM peeling, identifying suitable anatomical alternatives for plugging MHs remains challenging. Recently, several modern approaches have been introduced to address this issue, such as ILM plug harvested from the peripheral posterior pole, neurosensory retinal free flap, and capsular lens fragments [2,3,4,5,6]. Although these approaches offer promising alternatives, none has demonstrated consistent success in achieving complete anatomical closure or substantial improvement in visual acuity outcomes. Moreover, these techniques are often limited by procedural complexity, the requirement for multiple surgeries, or the unavailability of necessary tissues.

The human amniotic membrane (hAM) is the innermost layer of fetal membranes. It has a stromal matrix, a thick collagen layer, and an overlaying basal membrane with a single layer of epithelium [7]. The hAM is a basal membrane with antiangiogenic and anti-inflammatory properties. It functions as a framework for tissue repair, providing support to nearby cells and reducing apoptosis. Additionally, it secretes regenerative growth factors and suppresses the release of inflammatory mediators, namely interferon, interleukins, tumor necrosis factors, and platelet-derived growth factors [8].

Recent evidence suggests that inflammation plays a central role in the pathogenesis of several retinal diseases, including MH. Given its ability to dampen inflammation through its anti-inflammatory and secrete regenerative properties, it holds promise as an effective treatment option for refractory cases [8]. The use of hAM grafts for closing refractory macular holes was initially proposed by Rizzo et al. In their study involving eight patients, they achieved a 100% anatomical success rate with a mid-term follow-up of six months [9]. Additionally, the efficacy of hAM grafts has also been demonstrated in repairing retinal breaks, large macular tears associated with retinal detachment, and macular holes in highly myopic eyes with retinal detachment [9,10,11].

Findings mentioned above suggest that hAM grafts may offer a promising alternative in challenging vitreoretinal cases. Given these promising results, we aim to evaluate both the anatomical and functional outcomes of hAM grafts in the treatment of refractory macular holes.

## 2. Materials and Methods

This is a retrospective, cross-sectional study conducted among 11 patients affected by refractory or failed macular hole (FMH), treated with 23-gauge pars plana vitrectomy (PPV) and hAM graft transplantation by vitreoretinal surgeons at the JEC Eye Hospital (Jakarta, Indonesia) between December 2020 and December 2022. The sample size was limited due to the small number of patients who met the inclusion criteria and received this treatment during the study period. Written informed consent was obtained from all participants prior to the commencement of the study, in accordance with the principles outlined in the Declaration of Helsinki. The data collection, analysis, and reporting of this retrospective study were conducted following ethical approval by the Medical and Health Research Ethics Committee (MHREC), Faculty of Medicine, Public Health and Nursing, Universitas Gadjah Mada—Dr. Sardjito General Hospital.

Human amniotic membrane grafts were sourced from a certified local tissue bank (Batan Research Tissue Bank Pair, Jakarta, Indonesia). Dry, lyophilized hAM grafts sterilized using gamma irradiation were used. The tissue bank had no affiliation with this study.

The inclusion criteria were patients with FMH, with or without retinal detachment, who had previously undergone primary PPV, ILM peeling, and gas or silicone oil tamponade, with a follow-up period of at least 6 months. Patients were excluded if they had other coexisting retinal pathologies, such as age-related macular degeneration, uveitis, or retinopathy.

**Figure 1:**
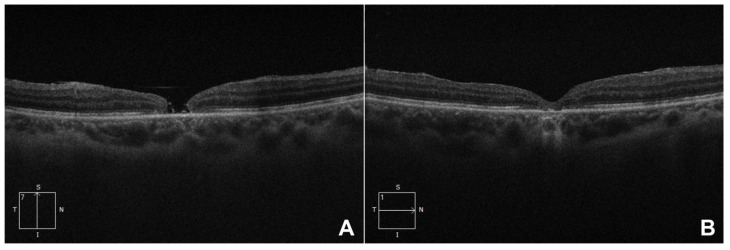
Preoperative (**A**) and 6-month postoperative (**B**) OCT scans of a patient who presented with a visual acuity of 1.477 logMAR and achieved a postoperative visual acuity of 0.523 logMAR.

Patients underwent examinations at baseline and at 3 and 6 months postoperatively. Each examination included best-corrected visual acuity (BCVA), slit-lamp biomicroscopy, intraocular pressure (IOP), and spectral-domain optical coherence tomography (OCT) to measure the inner minimum diameter of the macular hole. Figure 1 presents representative preoperative and 6-month postoperative OCT assessments from our patient cohort.

**Figure 2:**
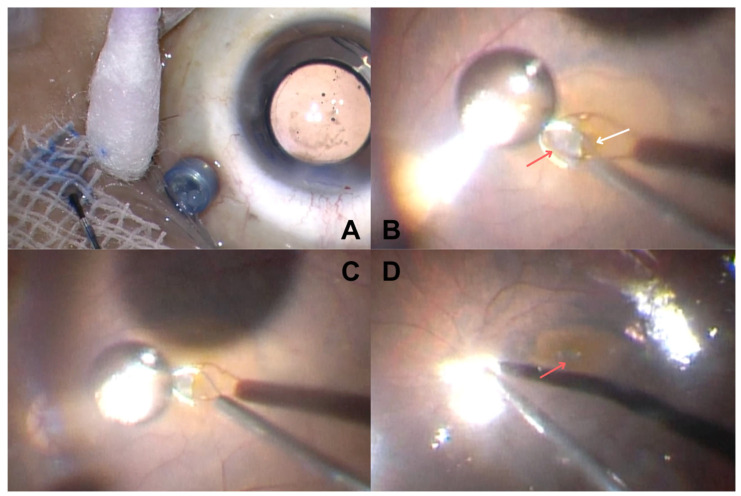
Intraoperative steps of hAM graft placement are shown: preparation of the graft stained with Brilliant Blue G to distinguish the epithelial and membrane sides (**A**), positioning the graft (*red arrow*) over the macular hole (*white arrow*) using a flex loop (**B**), and complete coverage of the macular hole by the graft (*red arrow in figure D*) (**C**,**D**).

BCVA was assessed using a Snellen chart and converted to the logarithm of the minimum angle of resolution (logMAR) for analysis. Intraoperative images of hAM graft placement are shown in Figure 2.

A visual acuity improvement was defined as an increase of 0.3 logMAR units or more, while a decline was defined as a decrease of 0.3 logMAR units or more. Postoperative adherence to prone positioning was monitored through patient self-report during follow-up visits. No objective measures were used to quantify compliance. Descriptive statistics were calculated, and the nonparametric Wilcoxon rank-sum test was used to analyze visual acuity improvements, as the data were not normally distributed. *p*-values < 0.05 were considered statistically significant.

## 3. Results

**Figure 3:**
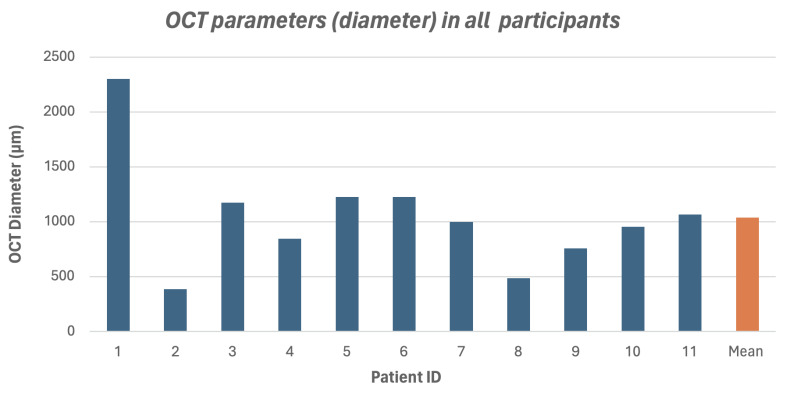
OCT parameter (diameter) in all participants.

**Figure 4:**
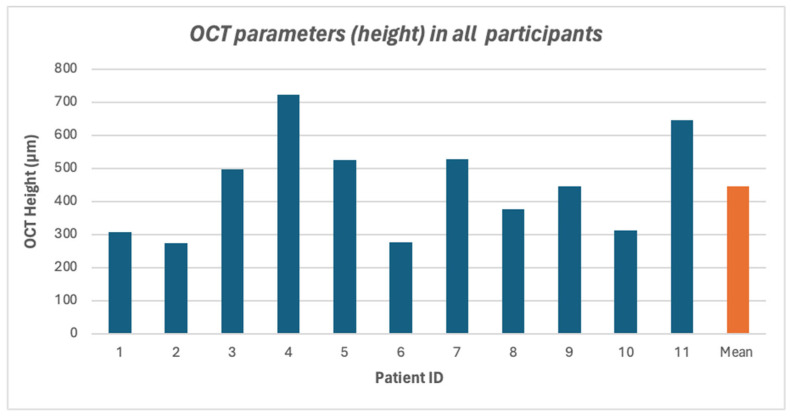
OCT parameter (height) in all participants.

Eleven eyes of 11 patients with full thickness macular hole (FTMH) who had already undergone primary 23-gauge PPV with complete ILM peeling were included in this study. Eight patients (72.7%) were male, and 3 patients (27.3%) were female. The mean age was 49.27 years (range 22–65 years). Two patients (18.2%) were phakic, while one patient (9.1%) and eight patients (72.7%) were aphakic and pseudophakic, respectively. The mean preoperative BCVA was 1.75 ± 0.73 logMAR (range 0.80–2.47 logMAR). Silicone oil was utilized in six patients, while sulfur hexafluoride (SF6) gas was employed in the remaining five. Table 1 summarizes the patients demographics and baseline clinical data. The mean macular hole diameter was 1037.09 ± 503.61 μm (range 384–2299 μm) as seen in Figure 3 and Figure 4. Details of preoperative OCT measurements are provided in Supplementary Table S1.

At the 6-month follow-up, complete anatomical closure of the macular hole was achieved in 9 of the 11 patients (81.82%). Two patients experienced hAM graft dislocation and required a second surgery involving repeat hAM transplantation. In both cases, successful closure was confirmed at the final follow-up. The first patient presented with a large macular hole, which may have compromised initial graft stability. In the second case, a history of traumatic macular hole was likely associated with underlying retinal pigment epithelium (RPE) defect, which may have impaired graft adherence.

**Figure 5:**
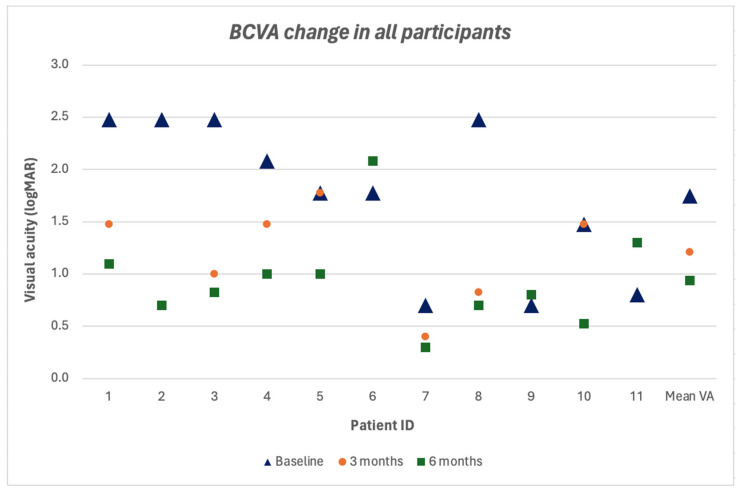
BCVA changes in all participants.

Shown in Figure 5, the mean BCVA improved by 0.537 logMAR at 3 months and by 0.808 logMAR at 6 months compared to baseline, with a statistically significant difference (*p* = 0.0207). Vision worsened by less than 0.3 logMAR units in one patient (9.1%), worsened by more than 0.3 logMAR units in two patients (18.2%), and in eight patients vision improved more than 0.3 logMAR units (72.7%). BCVA data across baseline, 3-month, and 6-month time points are shown in Supplementary Table S2.

## 4. Discussion

The treatment of refractory or failed MH is still a challenge for vitreoretinal surgeons. Among the many new techniques for treating refractory MH that have been proposed, the peeling of ILM from the periphery of the posterior pole, neurosensory retinal transplantation, and hAM graft transplantation are some of the techniques that have proven successful [2,9,12].

In our study, two patients (18.18%) needed another additional hAM graft transplant surgery after primary intervention. Several factors may contribute to successful resolution following the primary intervention. We believe patient-related factors greatly affected the success of MH resolution in these patients, particularly nonadherence to the recommended prone positioning, which led to graft displacement. After the second surgery and total adherence to our management protocols, these patients showed complete closure of their MHs.

In a study by Rizzo et al. [9], the hAM graft was placed under perfluorocarbon (PFC) without regard to which layer should be in contact with the RPE. It was inserted into the subretinal space followed by further filling of the PFC beyond the MH border, fluid-air exchange, and tamponade with SF6 or SO. All patients who received a gas injection achieved post-operative closure with an increase in mean BCVA from 20/800 to 20/50 at 6-month follow-up. The authors reported that the outer retinal band began to be integrated with the edges of the hAM graft on OCT examination [9]. Whether this indicates that the hAM graft stimulates retinal proliferation and visual improvement remains controversial. Meanwhile, Caporossi et al. paid particular attention to orienting the hAM as such that the chorion layer of the graft faces the RPE. They studied 16 eyes with axial myopia with refractory large FTMHs after ILM surgery. SF6 tamponade was used in 10 patients and air tamponade in 6 patients, in a prone position for 5 days for each patient. The increase in BCVA was moderate, with 68.75% of patients (*n* = 11/16) showing an increase in mean visual acuity from 20/200 preoperatively to 20/100 at follow-up 6 months after surgery. The closure rate was 93.8% (*n* = 15/16) [13]. In another study, Caporossi et al. also reported that at the 6-month follow-up, complete retinal reattachment with MH closure was found in all 10 eyes with high myopic macular hole associated with retinal detachment. MH closure was found at the 2-week follow-up in patients with silicone oil and at the 1-month follow-up in patients with C3F8 gas [11]. Our study results were comparable with previous studies in terms of MH closure.

Our study also found functional improvement as demonstrated by an overall improvement in post-operative BCVA. Our mean BCVA at 3-month and 6-month follow up demonstrated improvement with an increase of more than 0.3 logMAR units, from 1.747 ± 0.74 logMAR to 1.210 ± 0.51 logMAR and 0.939 ± 0.47 logMAR (range 2.079–0.301 logMAR), which was statistically significant (*p* = 0.0207). An increase in mean BCVA was also reported by Caporossi et al. In their study, 33 eyes with complete MH closure showed improved BCVA, while 3 other patients without MH closure (8.4%) had stable or worsening BCVA [14]. Morizane et al. also reported BCVA increase from 0.99 ± 0.25 to 0.57 ± 0.36 logMAR in 8 out of 9 patients that achieved macular hole closure [2]. Our findings were similar, although we encountered two graft dislocation cases (18.2%). Pooled data showed a 6% rate of graft dislocation in refractory MH studies, with a higher number of dislocations seen in dehydrated grafts (26%) compared to cryopreserved grafts (3%) [15]. The difference in dislocation rates may be explained by the thicker stromal structure of cryopreserved grafts, but further studies are needed to analyze how different grafts affect closure rate of refractory MH.

Complete anatomical closure after primary intervention was only observed in 9 out of 11 of our samples (81.82%), which differs slightly from Ferreira et al. Their study demonstrated complete resolution without any recurrences after a single intervention of hAM graft transplantation in all 19 eyes (100%) with a mean follow-up of 9  ±  3.87 months and a median of three lines of visual improvement [16]. Results from Ferreira et al. and our study were comparable to those reported in a study utilizing autologous retinal transplantation (ART), where complete anatomical closure was seen in 36 out of 41 eyes (87.8%) and mean visual acuity improved in 52.3% of eyes among those 36 eyes with anatomical closure [12]. However, ART is a complex procedure that poses limited practical application due to the need of specialized scissors, bimanual techniques, and graft transportation. Moreover, it has a greater potential for intra- and post-operative complications, with a pooled complication rate of 15% across multiple studies [17]. In contrast, hAM grafts are easy to obtain from the tissue banks. Compared to other MH closure substrates, such as ILM plugs, autologous retinal plugs, or capsular lens fragments, hAM grafts are easier to manipulate intraocularly.

This study has several limitations. As a retrospective analysis with a relatively small sample size and no control group, our findings are subject to selection bias and limited generalizability. The use of different tamponade agents (silicone oil and SF6 gas) without subgroup analysis may have introduced confounding variables affecting closure rates. Furthermore, adherence to postoperative positioning was assessed via patient self-report, which prevents objective evaluation of compliance. Additional prospective studies with larger cohorts, standardized protocols, and appropriate control groups are needed to more definitively assess the efficacy of hAM grafts for refractory macular holes. In-depth intraoperative and postoperative analyses may also help clarify the interaction between the graft and retinal tissue.

In summary, the use of hAM graft seems to be a viable and effective alternative for the treatment of refractory MH with good anatomical and functional results. However, further larger prospective controlled studies with larger samples are necessary to confirm our results.

## Figures and Tables

**Table 1:** Baseline characteristics of participants.

Characteristics	Participants (*n* = 11)
Sex	
Male	8 (72.7%)
Female	3 (27.3%)
Age group (Years)	
21–30	1 (9.1%)
31–40	2 (18.2%)
41–50	2 (18.2%)
51–60	4 (36.4%)
>60	2 (18.2%)
Baseline VA	1.75 ± 0.73
(logMAR, mean ± SD)	
Number of surgery	
1	9 (81.8%)
>1	2 (18.2%)
Lens Status	
Phakic	2 (18.2%)
Pseudophakic	8 (72.3%)
Aphakia	1 (9.1%)
Tamponade used in first surgery	
Silicone Oil	6 (54.6%)
Gas	5 (45.4%)
Comorbidities	
Diabetes mellitus	2 (18.2%)
Myopia	1 (9.1%)

VA, visual acuity; SD, standard deviation.

## Data Availability

The original contributions presented in this study are included in the article/Supplementary Material. Further inquiries can be directed to the corresponding author(s).

## Supplementary Materials

The following supporting information can be downloaded at: https://www.mdpi.com/article/10.3390/vision9020045/s1, Supplementary Table S1: Preoperative OCT measurement of macular hole; Supplementary Table S2: BCVA change in all participants.

## Abbreviations

The following abbreviations are used in this manuscript:

MH	Macular hole
ILM	Internal limiting membrane
hAM	Human amniotic membrane
AMT	Amniotic membrane transplantation
BCVA	Best-corrected visual acuity
IOP	Intraocular pressure
OCT	Optical coherence tomography
logMAR	Logarithm of the minimum angle of resolution
PPV	Pars plana vitrectomy
FMH	Failed macular hole
SF6	Sulfur hexafluoride
SO	Silicone oil
RPE	Retinal pigment epithelium
ART	Autologous retinal transplantation
PFC	Perfluorocarbon
