# Supplementary material for: Anatomical and Functional Outcomes of Human-Amniotic Membrane Graft in Refractory Macular Hole Cases
Source: Vision (Basel). 2025 May 22;9(2):45. doi: 10.3390/vision9020045 (PMC12197465; doi:10.3390/vision9020045)
Supplement: Supplementary file 1 [file vision-09-00045-s001.zip › vision-3570644-supplementary.pdf]

Supplementary Table 1. Preoperative OCT measurement of macular hole.

No. Patient	Diameter (µm)	Height (µm)
1	2299	308
2	384	275
3	1172	498
4	844	722
5	1226	526
6	1225	278
7	997	529
8	487	376
9	756	447
10	954	312
11	1064	647
**Mean**	**1037.09**	**447.09**
µm, micrometer		

Supplementary Table 2. BCVA change in all participants.

No. Patient	Baseline VA logMAR	VA at 3 Month logMAR	VA at 6 Month logMAR
1	2.477	1.477	1.097
2	2.477	0.700	0.700
3	2.477	1.000	0.824
4	2.079	1.477	1.000
5	1.778	1.778	1.000
6	1.778	2.079	2.079
7	0.700	0.400	0.300
8	2.477	0.824	0.700
9	0.700	0.800	0.800
10	1.477	1.477	0.523
11	0.800	1.301	1.301
**Mean**	**1.747**	**1.210**	**0.939**

VA, visual acuity; logMAR, logarithm of the Minimum Angle of Resolution
